# Organized Emergence of Multiple-Generations of Teeth in Snakes Is Dysregulated by Activation of Wnt/Beta-Catenin Signalling

**DOI:** 10.1371/journal.pone.0074484

**Published:** 2013-09-03

**Authors:** Marcia Gaete, Abigail S. Tucker

**Affiliations:** 1 Department of Craniofacial Development and Stem Cell Biology, King’s College London, London, United Kingdom; 2 Department of Anatomy, Faculty of Medicine, Pontificia Universidad Católica de Chile, Santiago, Chile; Laboratoire de Biologie du Développement de Villefranche-sur-Mer, France

## Abstract

In contrast to mammals, most reptiles constantly regenerate their teeth. In the snake, the epithelial dental lamina ends in a successional lamina, which proliferates and elongates forming multiple tooth generations, all linked by a permanent dental lamina. To investigate the mechanisms used to control the initiation of new tooth germs in an ordered sequential pattern we utilized the polyphodont (multiple-generation) corn snake (*Pantherophis guttatus*). We observed that the dental lamina expressed the transcription factor Sox2, a multipotent stem cell marker, whereas the successional lamina cells expressed the transcription factor *Lef1,* a Wnt/β-catenin pathway target gene. Activation of the Wnt/β-catenin pathway in culture increased the number of developing tooth germs, in comparison to control untreated cultures. These additional tooth germs budded off from ectopic positions along the dental lamina, rather than in an ordered sequence from the successional lamina. Wnt/β-catenin activation enhanced cell proliferation, particularly in normally non-odontogenic regions of the dental lamina, which widely expressed *Lef1,* restricting the Sox2 domain. This suggests an expansion of the successional lamina at the expense of the dental lamina. Activation of the Wnt/β-catenin pathway in cultured snake dental organs, therefore, led to changes in proliferation and to the molecular pattern of the dental lamina, resulting in loss of the organised emergence of tooth germs. These results suggest that epithelial compartments are critical for the arrangement of organs that develop in sequence, and highlight the role of Wnt/β-catenin signalling in such processes.

## Introduction

Polyphyodonty, the capacity for continuous tooth renewal, is displayed in most vertebrates, including amphibians, fish and reptiles. However, this capacity has been evolutionary lost in most mammals, which are restricted to diphyodonty, having two set of teeth (i.e. humans) or to monophyodonty, with one set of teeth (i.e. mice). The reduction in regenerative capabilities has been linked to a trade off between tooth number and tooth complexity and size [1].

**Figure 1:**
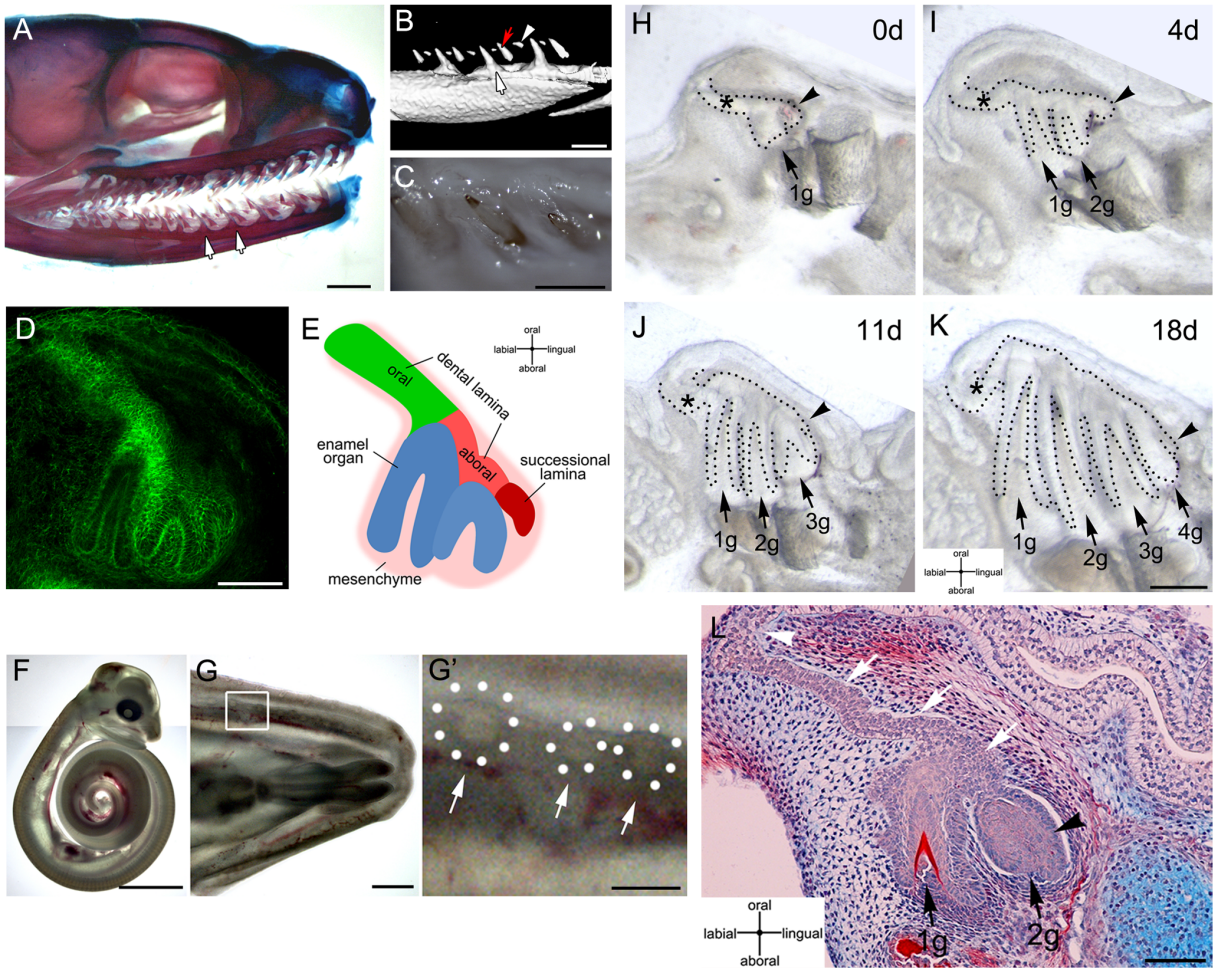
Slice organ culture of snake mandibles allows the formation of several generations of teeth. (A–C) Newborn corn snake (*Pantherophis guttatus*). (A) Alcian blue-Alizarin red skeletal staining of the head. (B) Mandible microCT scan. (C) Oral view of erupted teeth. (A–B) White arrows indicate functional teeth. (B) Red arrow and arrowhead point to replacement teeth. The arrowhead indicates the younger replacement tooth. (D) 3-day cultured corn snake dental organ. Fibrillar-Actin (green) is used to show the outline of the developing tooth germs. (E) Schematic of the regions of the dental organ represented in D. (F) 30-days post-oviposition snake embryo. (G) Mandible from embryo in C. (G′) Magnification of the framed area in G, dental organs can be identified throughout the mucosa indicated by arrows and dotted circles in the inset. (H–K) 18-day culture period. Several generations of teeth develop over this period. (L) Histology of dental lamina after 10 days in culture. (H–L) Black arrows: tooth germs; 1 g, 2 g, 3 g, 4 g, are 1^st^, 2^nd^, 3^rd^, and 4^th^ generations of teeth. Black arrowheads: successional lamina. White arrows: dental lamina. White arrowhead: origin of dental lamina from the oral epithelium. Scale bars: (A) 100 µm, (C,F) 1 cm, (G) 1 mm, (D,E) 500 µm, (G′,H–K) 250 µm, (L) 100 µm.

Teeth are derived from the oral epithelium and neural-crest derived mesenchyme [1], [2], [3]. They develop through key morphological stages, dental lamina, bud, cap and bell, according to the shape that the epithelium adopts. In the monophyodont mouse only one generation of teeth are formed, while in polyphyodont reptiles new generations are continuously formed throughout life [1], [3] and can be observed in a newborn snake (Figure 1A–C). Tooth development in polyphyodont reptiles is initiated by the formation of an epithelial dental lamina that grows into the developing jaw. In the case of the mandible, an epithelial bud appears on the labial side of the dental lamina and is surrounded by a condensed mesenchyme [3]. The dental lamina grows in a lingual direction and a group of cells at its free end, the successional lamina, proliferates. As the dental lamina elongates, the formation of several tooth generations are observed. In polyphodont snakes and lizards these generations are connected by the dental lamina into a regulated line of successively developing stages (Figure 1D, E). Tooth cusp shape and histo-differentiation occur during the cap and bell stages. Upon eruption into the oral cavity, the teeth become functional [3] (Figure 1A–C). General organ regeneration is the result of stem cells, so it is a fundamental question in oral biology to understand where dental stem cells are located in polyphyodonts and monophyodonts. In the gecko, the lingual side of the dental lamina, next to the successional lamina, houses BrdU label-retaining cells that express stem cell markers and it has been proposed as a putative niche for dental epithelial stem cells in the lizard [4]. In the mouse, the transcription factor *SRY (sex determining region Y)-box 2 (Sox2)* has being described as a marker of epithelial dental stem cells [5].

In polyphyodont models, teeth have to be induced in a highly coordinated manner to permit their proper development at regular intervals and with directionality, forming new tooth germs only on the lingual side of the dental lamina in the mandible. However, the molecular control of this repetitive and polarized tooth initiation, and how the induction of new teeth is regulated in this precise spatial-temporal sequence are not well understood.

Several signalling pathways have been shown to play important roles during tooth development. Among these, the evolutionary conserved Wnt/β-catenin pathway is a good candidate for controlling the induction and number of teeth in the different types of phyodoncy [2]. Wnt/β-catenin signalling acts by inhibiting the activity of GSK3β, which forms a complex containing Axin2 and adenomatous polyposis coli protein (APC). This inhibition allows the stabilization and nuclear translocation of β-catenin, inducing gene transcription of Wnt/β-catenin target genes via the LEF/TCF family of transcription factors. This pathway can control cell proliferation and cell fate during development [6], [7]. In the mouse, active Wnt/β-catenin signalling occurs throughout tooth development in a dynamic pattern [8]. An increase of Wnt/β-catenin signalling, caused by stabilisation of epithelial β-catenin or by loss-of-function of APC, produces abnormal dental invaginations and ectopic tooth formation, which increases the number of tooth germs into dozens of multiple small and ectopic teeth [9], [10], [11]. Conversely, epithelial deletion of β-catenin results in the inhibition of tooth development, decreasing the number of teeth [9].

Lymphoid enhancing factor 1 (*Lef1*) is a cell-type specific transcription factor that binds β-catenin. Its transcription is regulated by the activity of Wnt/β-catenin ligands and the Wnt/β-catenin pathway [12]. Epithelial up-regulation of *Lef1* using the *K14:Lef1* transgenic mouse allows the formation of extra-teeth in non odontogenic areas such as the vestibular lamina [13]. In a similar way, in *Epiprofin* knockout mice, which develop supernumerary teeth, *Lef1* is broadly expressed in tooth germs [14]. In contrast, *Lef1* mutant mice show an arrest of tooth development at the bud stage and an increase in apoptosis in the dental epithelium [15].

In animals that replace their teeth Wnt/β-catenin may play a role in replacement tooth initiation. In the diphyodont ferret, *Axin2* is expressed in the mesenchyme next to the successional dental lamina [16]. In contrast, the successional lamina of the monophyodont, bearded dragon, *Pogona vitticeps*, which does not produce additional teeth, does not express *Axin2* or *Tcf7* [3]. In the polyphyodont snake, *Python regius*, the Wnt ligands *Wnt10b* and *Wnt6,* and Wnt/β-catenin target genes *Axin2* and *Lef1* are expressed in the dental lamina, particularly in the successional lamina [17]. The active successional lamina of polyphyodonts is highly proliferative [4], [17], [18]. When GSK3β inhibitors are added to enhance Wnt/β-catenin signalling at early stages of dental lamina development, proliferation of the dental lamina is enhanced, mainly on the lingual side, increasing its size [4] and causing broad expression of *Lef1* in the dental lamina [17]. These experiments were performed at early stages to investigate the role of Wnt/β-catenin during initiation of the first tooth, however, it is not clear the long-term effects on tooth renewal or tooth number. For this, assessment of the function of Wnt/β-catenin in multi-generational stages of a polyphyodont model is necessary, to clarify the role of this pathway in the control of tooth number. Furthermore, the cellular mechanisms responsible for sequential tooth emergence and organization are still unknown.

Here, we describe the fate and expression of genes and proteins in the dental lamina at stages when multiple generations of teeth are developing in the polyphodont corn snake (*Pantherophis guttatus)*. This snake has been recently used in developmental studies [19], [20]. Using cultures of developing dental tissue we observed that Wnt/β-catenin overactivation affected the molecular and proliferative pattern of the dental lamina and increased the number of tooth germs, which appeared in ectopic positions along the lamina. Our results suggest a model in which Wnt/β-catenin signalling maintains a regenerative domain at the tip of the dental lamina that allows reiterative and restrictive spatial tooth initiation in polyphyodonts.

## Materials and Methods

### Pantherophis Guttatus Colony

The King’s College London genetically modified organisms (GMO) committee and the Home Office regulate all ethical issues related to the experiments performed. Adult *Pantherophis guttatus* snakes were maintained in a controlled environment with high standards of veterinary care within the King’s College London Biological Service Unit. All the animals are treated according to the refinement principles: respect, care and minimizing suffering. The euthanasia and manipulation procedures are according to ethical concepts that diminish animal pain.

Eggs were collected at laying and maintained on humid vermiculite at 30°C. After 28–35 days post-oviposition the snake embryos had reached an equivalent to stage 5 of African house snake development (*Boaedon fuliginosus*) [21]. *Pantherophis guttatus* and *Boaedon fuliginosus* are both from the family Colubridae, making this the closest match for our analysis.

MicroCT (computed tomography) scan was performed on newborn snake heads using a GE Locus SP microCT scanner. 3 D isosurfaces were generated by MicroView software (GE). Skeletal staining of newborn snake heads was performed in 70% ethanol, 0.3% Alcian Blue in 70% Ethanol, 0.1% Alizarin Red in 95% Ethanol and acetic acid in a 17∶1∶1∶1 proportion. The skulls were then cleared in KOH, KOH:glycerol and stored in glycerol.

### Tooth Culture

Snake mandibles were dissected out and cross-sectioned into 250 µm slices using a McIlwain tissue chopper (Mickle Laboratory Engineering Co., Ltd. UK). Slices containing complete dental organ with dental lamina and associated tooth buds were selected and cultured on transparent nucleopore filters (VWR) supported by metal grids at the surface of the medium as described [20], with minor differences: cultures was followed up to 18 days and the culture media used was Advanced DMEM/F12 (Invitrogen) supplemented with 1% penicillin/streptomycin, 1% Glutamax (Invitrogen). Slices were cultured at 30°C/5% CO_2_ changing the medium 3 times/week. For functional experiments GSK3β inhibitors were tested at different concentrations and the maximum concentration that did not affect the general growth of the culture was chosen: 10 mM LiCl and 30 µM BIO (GSK3β inhibitor 6-bromoindirubin-3-oxime, EMD Biosciences) were added to the media. DMSO was added to BIO-control cultures. Experimental and control treated cultures were either taken from the same slice divided into left and right halves or by using consecutive slices to make sure the samples were age and positioned matched. A minimum of 5 dental organs from two snake specimens were used for each treatment. Due to the difficulty of identifying rudimentary from ectopic teeth all tooth/tooth germs observed were counted and the difference in number, compared to day 0, was assessed.

In addition to the slice culture method, snakes mandibles were dissected out and divided into left and right halves and proximal and distal segments and cultured as a block of tissue. The left and right equivalent halves were used for experimental and control groups. Dental families were analyzed matching them in pairs with similar proximo-distal positions of the mandible from the contralateral side.

### Fate Mapping

For fate mapping, DiI (cell tracker CM-DiI, C-7000, Molecular probes) was resuspended in 100% ethanol and injected into the slices. The position of the DiI label was recorded on different days post-labelling.

### Histology and *in situ* Hybridization

For histology and *in situ* hybridization, snakes cultures were fixed in 4% PFA for 2 hours at room temperature, washed in PBS and dehydrated in methanol. For histology, samples were incubated in isopropanol, cleared in 1,2,3,4 tetrahydronaphtalene and embedded in paraffin. 8 µm sections were stained with Pico-Sirius Red, haematoxylin & Alcian Blue 8 GX. For whole mount *in situ* hybridization, samples were rehydrated, permeabilized in detergent mix (1% IGEPAL, 1% SDS, 0.5% Deoxycholate, 50 mM Tris pH 8, 1 mM EDTA, 150 mM NaCl) for 15 minutes, and incubated in pre-hybridization mix (50% formamide, 5×SSC pH 4.5, 0.1% Tween-20, 2% BBR (Boehringer, 1096176), 250 µg/ml tRNA, 100 µg/ml heparin at 65°C for 2 hours. Samples were hybridized in hybridization mix: 1 µg/ml of the *Lef1* probe in pre-hybridization mix overnight at 65°C. Samples were washed in Solution X (50% formamide, 2X SSC pH 4.5, 1% SDS) for 2 hours, washed in MABT 1 hour, blocked in 20% lamb serum and 2% BBR in MABT for 1 hour and incubated with 1∶2000 anti-DIG Alkaline Phosphatase antibody (Boehringer, 1093274) overnight at 4°C in blocking solution. Samples were washed in MABT for 2 hours and incubated in NTMT (100 mM Tris-HCl pH 9.5, 50 mM MgCl_2_, 100 mM NaCl, 0.1% Tween 20) pH9.5 for 30 minutes. The colour reaction was developed in 3.5 µl/ml BCIP and 4.5 µl/ml NBT in NTMT at room temperature. The samples were then fixed in 4% PFA and transferred to glycerol.

### Immunofluorescence

Whole-mount inmunostaining against Sox2 was performed as described [22]with minor modifications. Cultured slices were fixed in 4% PFA 2 hours at room temperature, permeabilized for 45 minutes in PBS containing 0.5% Triton X-100 (PBS-Tr), incubated in 0.25% trypsin for 15 minutes on ice, washed in PBS-Tr and incubated in blocking solution (PBS-Tr containing 10% goat serum and 1% DMSO) for 2 hours. Samples were incubated with the primary antibody rabbit polyclonal anti-human Sox2, which cross-reacts with several mammalian species (Cell Signalling 2748 s) and gives a consistent signal in *Xenopus* [22], or rabbit polyclonal anti-phospho-Histone H3 (pH3) (Upstate 06-570) in blocking solution overnight at 4°C and washed 2 hours in PBS-Tr. Samples were then incubated in the secondary antibody AlexaFluor® 568 goat anti rabbit for 2 hours and washed for 2 hours in PBS-Tr. For double labelling of pH3 and Fibrillar-Actin, whole mount inmunostaining against pH3 was followed by incubation in Alexa Fluor® 488 phalloidin (Invitrogen A12379) for 3 hours. DNA was stained with Hoechst stain solution (Sigma H6024) for 3 hours and mounted in *Mowiol®* 4–88 mounting media (Polyscience).

### Image Processing Quantification and Statistics

Slice cultures were photographed during the culture period using a Leica MZ FLIII Stereoscope. Samples with immunofluorescence and DiI labelling were scanned on a confocal microscope (Leica SP5 laser scanning confocal microscope). The number of tooth germs were assessed using the morphological shape of the epithelium and condensation of the surrounding mesenchyme in Z-stacks or histological sections. Some samples were used for both, *in situ* hybridization and immunofluorescence. Images were processed using Adobe Photoshop*®* and ImageJ (NIH). When needed, smart sharpen filter, hue-levels and despeckle plug-in was applied in equal amount in control and treated cultures. For quantification of volume and mitotic index in the total dental organ, 6–8 optical sections from z-stacks were used for each sample and quantified using Volocity Software (Perkin Elmer). For the mitotic index the percentage of pH3^+^ nuclei over the total amount of nuclei positive for Hoechst was determined. Abercrombie’s correction was applied in nucleus quantification. For regional pH3^+^ cell counting, a specific plane including the successional lamina and the rest of the dental organ was selected. Areas were delimitated into oral dental lamina (dental lamina without association to a tooth), aboral dental lamina (dental lamina associated to a tooth) and successional lamina (as a free edge of dental lamina or as a part of outer enamel organ of younger tooth germs). Afterwards, cell counting was performed using the ImageJ (NIH) cell counter plug-in. Results were plotted and analyzed by t-test for cell counting and by Wilcoxon matched pairs test for the number of tooth germs using Graph Pad Prism 5 software*®*. Error bars are Standard Error of the Mean (SEM). One (*) and two (**), asterisks correspond to p<0.05 and p<0.01 respectively.

### RT-PCR and Cloning

RNA was isolated from stage-9 dental organ using an RNA easy plus kit (Qiagen). Reverse transcription was performed using M-MLV Reverse Transcriptase (Promega). *EF1*α and *Lef1* transcripts were amplified by PCR from *Pantherophis guttatus* intestine and dental organ cDNA using degenerated primers designed empirically comparing sequences from *Homo sapiens, Gallus gallus, Xenopus laevis* and *Anolis carolinensis.* PCR fragments were cloned into pCRII vector (TOPO TA cloning kit, Invitrogen) and sequenced to confirm the identity of the fragments. *EF1*α and two *Lef1* isoforms with high protein sequence identity to vertebrate species were cloned and deposited on GenBank (Table 1). For *in situ* hybridization we mixed probes for simultaneous identification of both isoforms. Internal primers from: *EF1*α cloned sequence, *Axin2* (GenBank # EU196456.1) and *Lnfg* (GenBank # EU196458.1) were designed to perform RT-PCR. *EF1*α primers were: Fw 5′-GGAAGGTCACTCGTAAAGATGG-3′ and Rv 5′-GAACTGTACCAATCCCACCAAT-3′. Axin2 primers were: Fw 5′-CATTGTCTCCAAGCAACTCAAG-3′ and Rv 5′-AAGCATTTTCCTCCATCACAGT-3′. *Lfng* primers were Fw 5′- AAATTGCTCTCCAGCTATGCTC-3′ and Rv 5′-CAGTAATACGACTCACTATTA TGCCATAACTCAAAGTGACCTG-3′.

**Table 1:** *Pantherophis guttatus* cDNAs cloned in this study.

*P. guttatuts gene*	cDNA length (bp)	Protein sequence identity (%) - Protein sequence coverage (%) with				GenBankaccession #
		*Homo sapiens*	*Gallus gallus*	*Xenopus laevis*	*Anolis carolinensis*	
*Lef1 isoform 1*	1140	95–93	*	*	94–95	KF042297
*Lef1 isoform 2*	1056	95–93	95–92	94–86	*	KF042298
*EF1α*	1088	78–98	78–99	78–96	78–99	KF042299

* No isoforms described.

## Results

### Organ Slice Culture Permits Multigenerational Tooth Development to be Followed in the Snake

In order to illustrate the pattern of tooth replacement in the corn snake (*Pantherophis guttatus)*, we examined the dentition in the newborn snake using skeletal staining, microCT scan and oral examination. We observed several functional teeth ankylosed to the mandible (see white arrows in Figure 1A, B). Some teeth appeared mineralized but had not yet ankylosed to the bone (see red arrow in Figure 1B). The most lingual replacement teeth appeared mineralized only at the tip of the cusp (see arrowhead in Figure 1B), demonstrating the production of new teeth towards the lingual side of the mandible. Functional teeth were observed erupted into the oral cavity (Figure 1C). To investigate the formation of several dental generations, we examined dental organs of snake embryos at different days-post-ovoposition to identify the stages of successional lamina development. We classified snake embryos according to their body characteristics using a staging table for the African house snake [21]. From stage 5 (Figure 1F) we observed that all dental families had at least one generational tooth and a recognizable successional lamina (Figure 1D,E). The location of each group of tooth germs could be identified in developing mandibles as translucent circles (see Figure 1G and dotted circles in Figure 1G′). Mandibles were cross-sectioned and cultured using the slice organ culture method, which allows visualisation of the developing tooth germs (Figure 1H–K). During an 18-day culture period, three to four new generations of sequential teeth developed (see arrows in Figure 1H–K). The dental lamina and successional lamina were clearly recognizable (asterisks in Figure 1H–K indicate oral dental lamina; arrowhead indicates successional lamina). When sectioned for histology the cultured dental organs showed developing tooth germs with formation of dentine (Figure 1L). The dental lamina bends in an acute angle from the oral epithelium on its lingual side (see white arrowhead in Figure 1L). The dental lamina grew into the mesenchyme of the jaw and connected the tooth germs in a chain (see white arrows in Figure 1L). The successional lamina was localized at the most lingual side of the dental lamina (see black arrowhead in Figure 1L). Teeth appeared to emerge from the successional lamina region and developed connected to the dental lamina.

### A Subset of Successional Lamina Cells are Retained within the Lamina as New Generations of Teeth Appear

**Figure 2:**
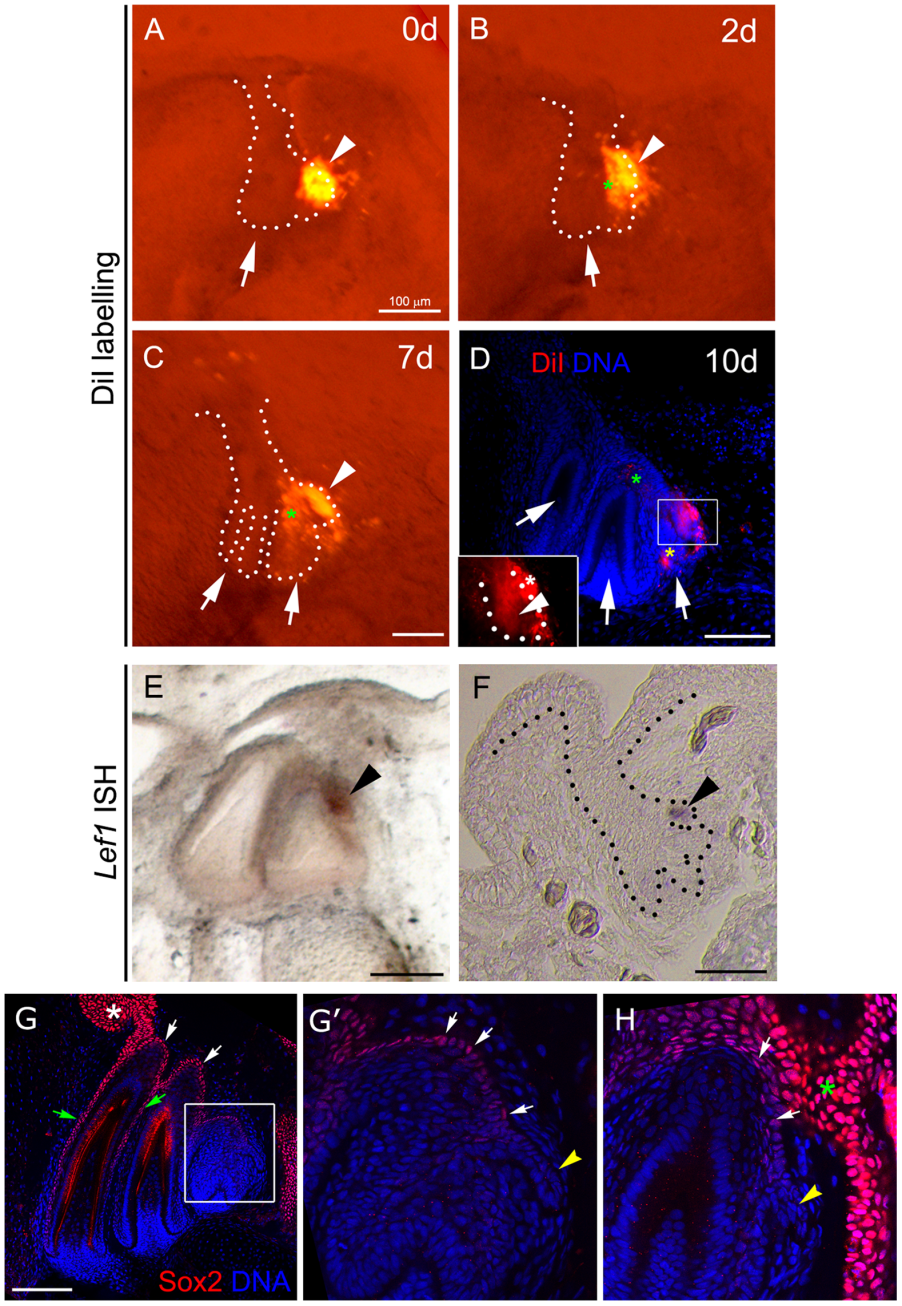
Fate and expression pattern of the dental lamina in the snake. (A–D) 10-day culture of the snake dental organ labelled with DiI. (A, B, C) fluorescence microscopy and (D) confocal optical section. (A) 0-day culture. DiI labelling was performed at the region of the successional lamina (arrowhead). (B) 2-day culture. (C) 7-day culture. (B,C) The label remains in the region of the successional lamina (arrowhead) and in the forming second tooth (green asterisk). (D) Day 10. Magnification in D shows the framed area. Label is present in the second (green asterisk) and third (yellow asterisk) generation tooth germs and is retained in the successional lamina (arrowhead) and adjacent mesenchyme (white asterisk). (E–F) *Pantherophis guttatus Lef1* mRNA is located in the successional lamina region (arrowhead), as observed by whole mount (E) and section (F) *in situ* hybridization. (G,G′,H) Sox2^+^ cells are found in the oral (asterisk) and aboral dental lamina (white arrows) and outer enamel epithelium (grey arrows), but are excluded from the successional lamina (yellow arrowhead in G′, H). (G′) magnification of the region indicated in G. (H) Lingual plane showing the connection of the dental lamina with the oral epithelium. Scale bars: 100 µm.

To examine whether the successional lamina contribute to the formation of new generations of teeth, the fate of this tissue was studied by lineage tracing experiments. The dental epithelium and adjacent mesenchyme were labelled with DiI and followed over 10 days in culture. In 6 out of 30 cultures the DiI labelled the region of the successional lamina at Day 0 (Figure 2A). After 2 and 7 days, the DiI label in the region of the successional lamina was maintained in the successional lamina (see white arrowhead in Figure 2B,C) with label also observed in the newly formed second generation tooth germ (see green asterisk in Figure 2B,C). After 10 days the second generation had labelled cells and a further third generation tooth germ had emerged which also contained some labelled cells (see green and yellow asterisk in Figure 2D respectively). The successional lamina and the adjacent mesenchyme remained labelled (see white arrowhead and white asterisk in Figure 2D inset). Throughout the culture period, DiI label was retained at the tip of the successional lamina and in the adjacent mesenchyme, this suggests that the two populations of cells remain together during the culture period. Therefore, these results indicate that a portion of the cells in the successional lamina remain in the lamina during the production of new tooth generations.

### Sox2 Positive Cells are Present in the Dental Lamina, but not in the *Lef1*-expressing Successional Lamina

We next explored the molecular phenotypes of the dental and successional lamina during the formation of multiple generations of teeth. Previous studies on *Pythons (P. Sebae)* have demonstrated that *Lef1* is expressed at the free end of the dental lamina as the first tooth is generated [17]. We aimed to assess whether *Lef1* is expressed and remains restricted to the successional lamina over multiple tooth generations in *Pantherophis guttatus.* We cloned *Pantherophis guttatus Lef1,* which displays high conservation with other species (Table 1). By *in situ* hybridization, we observed *Lef1* was strongly expressed in the region of the successional lamina in cultured dental organs (Figure 2E,F), indicating maintained expression over several generations of tooth development.

Due to the snake’s ability to constantly renew its teeth, we expected to find a source of epithelial stem cells in the dental organ. Sox2 has been recently identified as a specific dental epithelial stem cell marker of mouse dental epithelium [5], we therefore investigated Sox2 expression during snake tooth development in culture. By immunofluorescence we detected Sox2^+^ cells along the oral epithelium, dental lamina (see asterisk and white arrows in Figure 2G) and outer enamel epithelium (see green arrows in Figure 2H). The number of Sox2^+^ cells in the dental lamina decreased accordingly as they approached the successional lamina, where Sox2 negative cells were observed (see zoom in Figure 2G′, compare white arrow with yellow arrowhead). On superficial planes, we observed a continuous connection of the Sox2^+^ lamina and the oral epithelium (Figure 2H). Sox2 is therefore expressed in the oral and dental epithelium of snakes, with the exception of the successional lamina in which *Lef1* expression is observed.

### Prolonged Inhibition of GSK3β in Snake Dental Organ Cultures Activates Wnt/β-catenin Signalling, Increasing the Number of Tooth Germs and Altering the Morphology of the Dental Lamina

**Figure 3:**
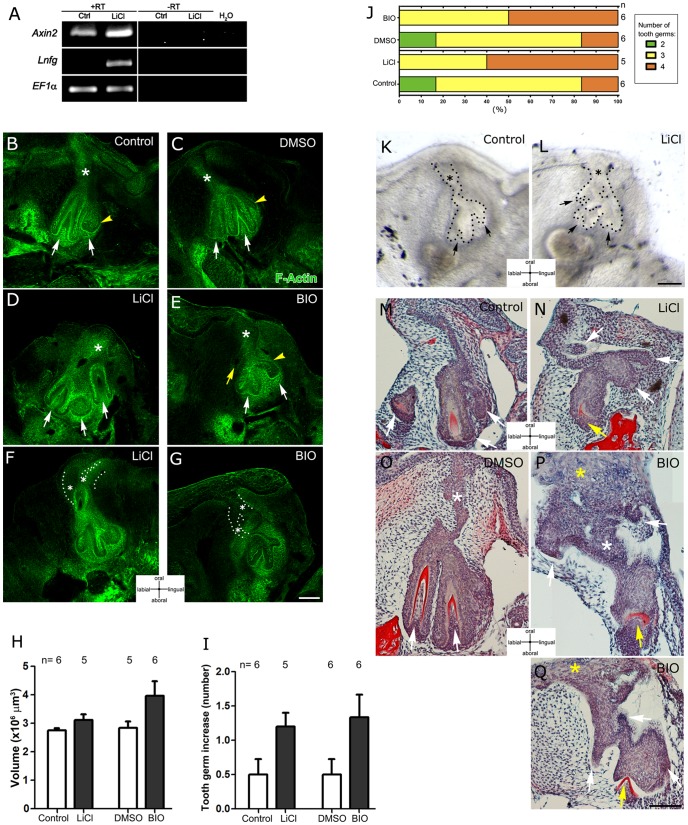
GSK3β inhibitors induce Wnt/β-catenin overactivation and increase the number of tooth germs in the snake. (A) RT-PCR analysis of *Axin2* and *Lunatic fringe (Lnfg)* mRNA levels of the dental organ indicating *Axin2* and *Lnfg* are up-regulated after LiCl treatment. *EF1α* was used as a loading control. (B–G) F-Actin detection after 3 days of culture in (B) Control, (D,F) LiCl, (C) DMSO and (E,G) BIO. Asterisks: dental lamina, White arrows: tooth germs; yellow arrowhead: successional lamina. (H) The volume of the dental organ does not change after GSK3β inhibitor treatment. (I) Number of new tooth germs that appeared during 3 days of treatment and (J) percentage of the number of tooth germs in 3-day snake cultures after treatment, n = number of slices analyzed. (K,L) Morphology of dental organs after 7 days of control culture (K) and LiCl culture (L). Asterisks: dental lamina, Black arrows: tooth germs. (M–Q) Histology of explanted dental organs after 10 days in culture: (M) control, (N) LiCl treatment. (O) DMSO control and (P,Q) BIO treatment. White arrows: tooth germs and epithelial projections. Yellow arrow: dysmorphic tooth germ. White asterisks: dental lamina. Yellow asterisks: acanthosis in dental lamina. Scale bars: 100 µm.

To understand the role of Wnt/β-catenin signalling during dental development, we performed gain-of-function experiments in culture. For Wnt/β-catenin activation, GSK3β was inhibited using LiCl or BIO. BIO-control cultures were treated with DMSO. For LiCl treatment, Wnt/β-catenin activity was assessed by RT-PCR of *Axin2*, a readout of the Wnt/β-catenin pathway [8], and *Lfng (lunatic fringe),* which is up-regulated indirectly by the Wnt/β-catenin pathway [23]. *Pantherophis guttatus EF1*α was cloned (Table 1) and used as loading control. *Axin2* mRNA levels increased and *Lfng* mRNA started to be observed in dental organs with LiCl treatments, indicating stimulation of the Wnt/β-catenin pathway (Figure 3A).

The number of tooth germs that developed in culture was followed for up to 10 days. At 3 days some cultures were fixed and Fibrillar-Actin was detected using phalloidin to outline the developing tooth germs. A range from 2 to 4 tooth germs were identified. An increase in the number of tooth germs (compared to the number of tooth germs observed at day 0) was observed in treated LiCl and BIO cultures compared to controls (see arrows in Figure 3B,C and compare with Figure 3D,E). On average, the number of new tooth germs that appeared over the 3-day period from day 0 in treated cultures was approximately 1.2; while in control cultures tooth number only increased by 0.5 (Figure 3I). Therefore, less than 20% of the control samples contain 4 teeth while after GSK3β inhibitor treatment this percentage increased to between 50–60% (Figure 3J).

To rule out any problems caused by the slicing technique, the experiments were then repeated using blocks of mandibular tissue in culture (Figure S1A). Using this technique the tooth germs could not be observed during the culture method and development was slower than that observed in slice culture. However, as with the slices, inhibition of GSK3β resulted in significantly more tooth germs forming compared to untreated tooth germs from the same proximo-distal position on the contralateral side of the jaw (Figure S1B–D).

In GSK3β inhibitor treated cultures, a range of alterations to the morphology and spatial organisation of the dental organ were observed using both techniques. In control cultures the new generations of teeth appear sequentially on the lingual side of the mandible (Figure 3B, C, Fig. S1B). All teeth and tooth germs developed with a space in between them (Figure 3B,C). However, in LiCl and BIO treated cultures a crowd of dental organs was observed with a minimum gap between them (Figure 3D,E). In some cases, tooth development was no longer restricted to the lingual side, with tooth germs observed budding off from other areas of the lamina (see yellow arrow in Figure 3E and Fig. S1C). The developing teeth appeared smaller, and although the number of teeth increased the volume of the intact dental organ did not significantly change (Figure 3H).

GSK3β inhibitors also affected the development of the dental lamina, which appeared shorter and wider compared to controls (See asterisk in Figure 3B,C, compare with Figure 3 D,E). Interestingly, in many cases double epithelial projections from the oral epithelium, which appeared to form secondary dental laminas, were observed in treated cultures (LiCl 3/5; BIO 3/6, Figure 3F,G). No similar secondary laminas were observed in untreated control cultures, however, some dual laminas were also observed after DMSO treatment, indicating that even at low levels DMSO can cause changes in morphology (Control 0/6; DMSO 2/6).

After 7 days of culture, multiple small tooth germ-like structures appeared clustered around the dental lamina (see arrows in Figure 3L), which was wider and shorter than controls (compare asterisk in Figure 3K,L). After 10 days of culture, a variety of alterations were observed. Most prominent was the detection of multiple epithelial projections throughout the dental lamina and in many cases a complete loss of normal morphology (Figure 3N,P,Q, compare with Figure 3M,O). After this prolonged treatment the tooth germs that had formed had defects in dental papilla formation, characterised by a shallow V-shaped deposition of dentine indicating a defect in the correct invagination of the epithelium (see yellow arrows in Figure 3N,P,Q, compare to Figure 3M,O). In BIO treated cultures, cells in the oral epithelium were enlarged and fused to the dental lamina. Hyperplasia and hypertrophy of the oral epithelium and dental lamina were observed concomitant with signs of acanthosis, thickening of basal layers (see yellow asterisk in Figure 3P,Q). Long-term inhibition of GSK3β therefore has deleterious effects on tooth morphology and differentiation.

Together these observations indicate that the number of tooth germs increased in GSK3β inhibited cultures, and that these additional tooth germs developed ectopically without proper polarization, suggesting defects in spatial tooth emergence.

### GSK3β Inhibition Expands the Domain of Expression of *Lef1*

**Figure 4:**
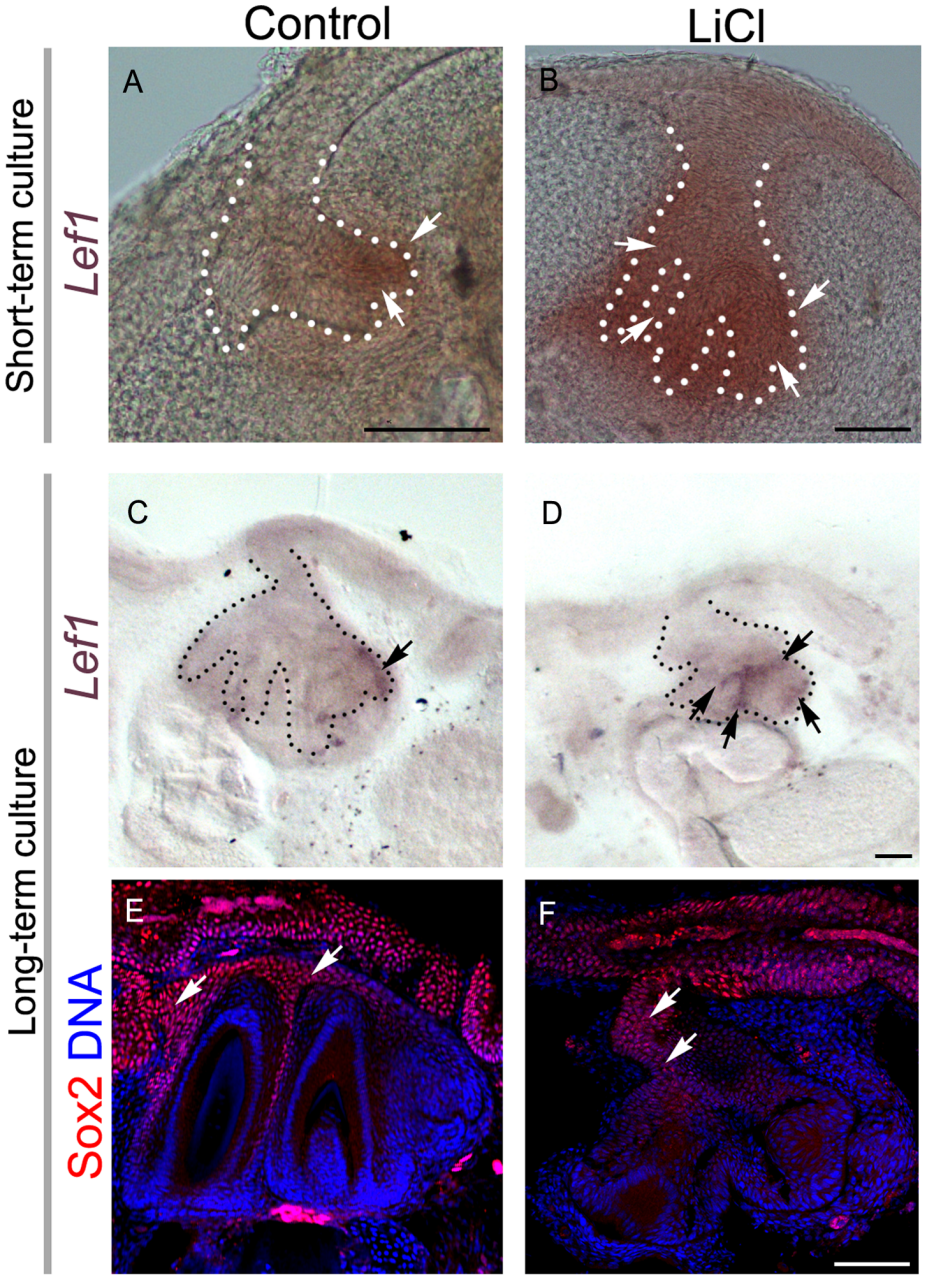
Treatment with LiCl alters the expression pattern of the dental lamina. (A–D) *Lef1* expression in 3-day culture (A, B) and 10-day culture (C–D) assessed by *in situ* hybridization. (A,C) Polarized pattern of expression of *Lef1* in control cultures. (B,D) loss of localized expression of *Lef1* after treatment with LiCl. (E,F) Sox2 immunofluorescence of 10-day culture in (E) control and (F) LiCl treatment. Arrows highlight the positive signal. Scale bars: 100 µm.

The changes in the morphology of the dental lamina in dental organs treated with GSK3β inhibitors prompted us to assess if the molecular behaviour of the dental lamina was also altered. For this, analysis of *Lef1* gene expression on LiCl treated cultures was performed. In long-term cultures exposed to LiCl, the expression domain of *Lef1* was expanded and the lingual polarization was lost (Figure 4D), while in control cultures *Lef1* was restricted to the successional lamina region (Figure 4C). GSK3β inhibition impaired tooth development such that the exact morphology of the dental organ was difficult to address in these long-term cultures. We therefore analysed short-term cultures in which the morphology of the dental organ were comparable. In controls, epithelial *Lef1* expression was restricted and polarized to the successional lamina region, however, it was expanded and non-polarized in the epithelium of LiCl treated cultures (Figure 4A,B).

To investigate the effect of the loss of the restriction of *Lef1* expression on the identity of the dental lamina, the expression of Sox2 was analysed. In LiCl treated cultures Sox2 expression was restricted to the dental epithelium close to the oral surface, the oral dental lamina, but without the elongated line toward the successional lamina as observed in controls (Figure 4E,F).

### GSK3β Inhibition Increases Epithelial Cell Proliferation within the Dental Lamina but has Limited Impact on Dental Mesenchyme Proliferation

**Figure 5:**
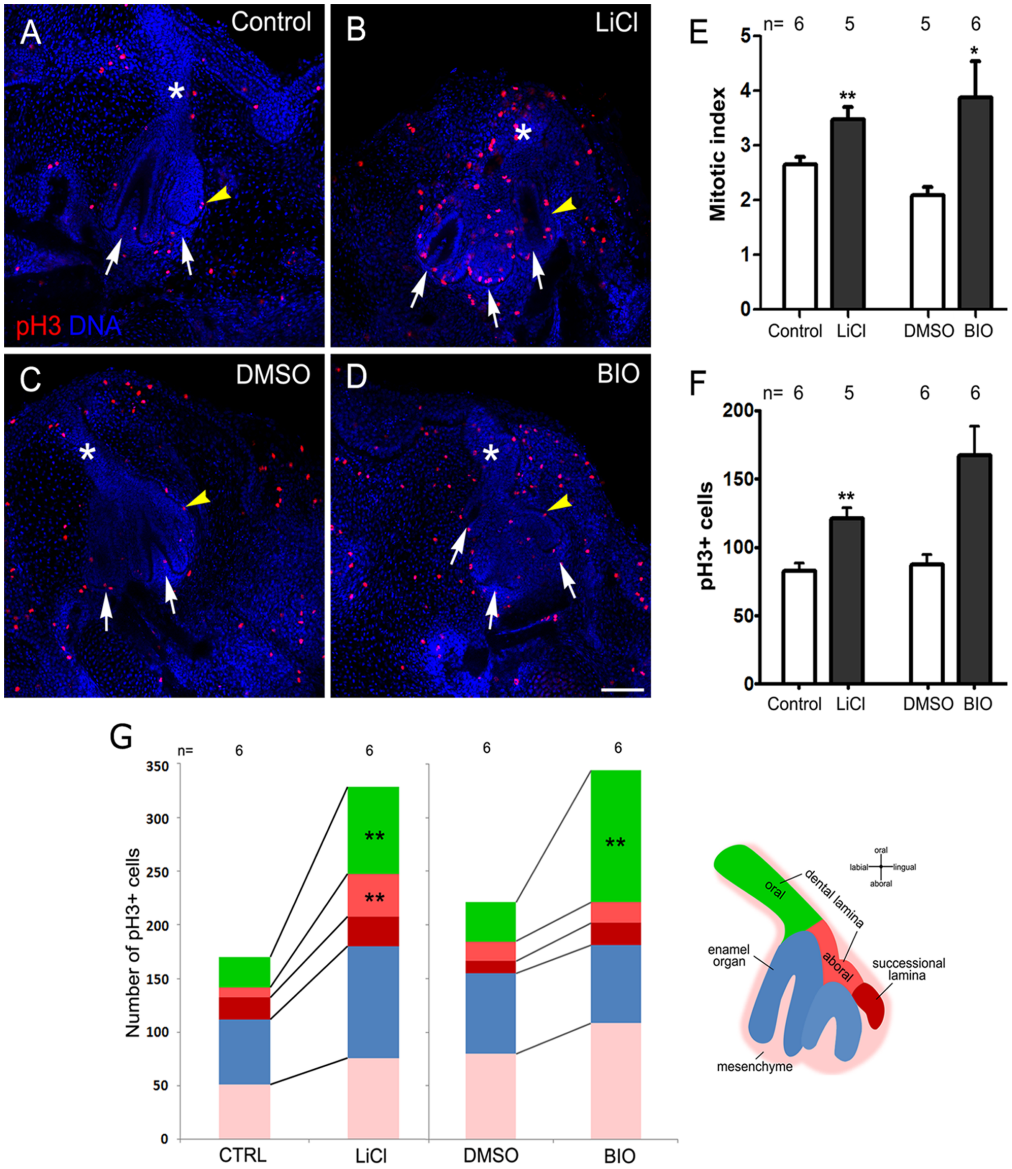
GSK3β inhibitors increase the proliferation of the dental lamina. (A–D) Immunofluorescence against phospho-Histone H3 (pH3) (red) in explants after 3 days of culture. DNA (Hoechst) is in blue. LiCl increases the number of pH3^+^ cells in the dental organ and the dental lamina appears wider in treated cultures (asterisk in B,D compared to A,C). (E, F) Quantification of the samples represented in A–D showing (E) increase of pH3^+^ cells and (F) increase in mitotic index after LiCl and BIO treatment, n = number of slices analyzed. (G) Distribution of the number of pH3^+^ cells along the different regions of the dental organ represented in the scheme. Significant differences were found in the dental lamina after LiCl and BIO treatment. Scale bar: 100 µm.

To understand the mechanisms triggered by GSK3β to regulate the number of tooth germs, we evaluated the effect of GSK3β inhibition on cell proliferation. For this, we performed whole-mount immunofluorescence against phospho-Histone H3 (pH3) in 3 days culture slices (as represented in Figure 3). Nuclear staining was confirmed by colocalization with DNA labelling. In control cultures we observed pH3^+^ cells in the epithelium and mesenchyme of the dental organ, which increased with GSK3β inhibitor treatment (Figure 5A–D). We quantified this result and the number of pH3^+^ cells in the entire dental organ significantly increased in LiCl and BIO treated cultures compared to the controls (Figure 5E). The mitotic index also significantly increased at a similar rate, confirming that proliferation increased the total number of cells (Figure 5F). However, despite this the volume of the dental tissue remained the same at this culture time point (Figure 3H), a thickening and shortening of the dental lamina was in evidence (Figure 3D,E, Fig. S1B,C).

We aimed to address whether the proliferation induced by GSK3β inhibition could be related to the change in the morphology of the dental organ. For this, we assessed which region of the dental tissue was most responsive to drug treatment. We quantified pH3^+^cells within several compartments of the dental organ: oral dental lamina, aboral dental lamina, successional lamina, enamel organ and the underlying dental mesenchyme (Figure 1E). When we compared LiCl and BIO treated cultures with controls, we observed the number of pH3^+^ cells preferentially increased in the oral dental lamina, the region of the dental lamina that is closest to the oral epithelium, and in the adjacent aboral dental lamina (Figure 5G). Interestingly, no significant change in proliferation was evident in the enamel organ or dental mesenchyme (Figure 5G). Taken together these results indicate inhibition of GSK3β increased proliferation of the snake dental organ preferentially in the dental lamina.

## Discussion

Snakes and other polyphyodonts have the capacity to regenerate their teeth constantly, and as such, polyphyodonts are excellent models for the study of organ renewal. A lack of genetic tools, however, has limited our understanding of these processes in such non-traditional models. Here, we have demonstrated a successful organ culture method to allow functional tests of organ regeneration over long periods. Using this method, we have evaluated the effect of Wnt/β-catenin overactivation via GSK3β inhibition in snake dental organ cultures. We have observed an alteration in proliferation and in the molecular patterning of the dental lamina, linked with an impairment of the sequential emergence of tooth germs.

### Dental Epithelium Compartments Express Different Molecular Markers

Organ regeneration in adult tissue is typically controlled by stem cells, which are defined by their ability for self-renewal and differentiation. Stem cells divide slowly and can form both a stem cell and a daughter cell. The daughter forms a transit amplifying progenitor cell that proliferates quickly for a limited number of divisions, giving rise to several differentiated cells. In regenerative organs, stem cells and their progeny are compartmentalized [24], [25]. Interestingly, the dental lamina has been proposed as a source of epithelial stem cells in gnatosthomes [26], and in this tissue some compartments can be identified.

We have observed that tooth development in snakes is achieved by local and molecularly defined epithelial cells, with the oral epithelium and oral dental lamina containing Sox2^+^ cells. In many organs, such as hair, pituitary gland and mammalian dental epithelium, stem cells express Sox2 [5], [27], [28]. The Sox2^+^ cells in the lingual aboral dental lamina in snakes coincide with the localization of putative dental stem cells in the polyphyodont gecko, which houses BrdU label-retaining cells and express stem cell markers [4]. This suggests that the Sox2^+^ cells in the snake dental lamina may be putative stem cells. In addition, Sox2^+^ cells were also found more widely in the snake oral epithelium, which is connected to the dental lamina epithelium. A similar widespread expression of Sox2 was observed in the mouse at early stages of development, with a later restriction of positive cells to the dental epithelium [5]. Sox2 expression is not present in the successional lamina in which *Lef1* is expressed. These findings are in agreement with previous works that demonstrate Wnt/β-catenin signalling pathway inhibits Sox2 expression [29]. Sox2^+^ cells in the lingual side of the aboral dental lamina may form progeny that end up in the successional lamina and switch off Sox2.

In several organs, *Lef1^+^* cells are located in specific regions. In the hair follicle *Lef1*expression is confined to cells in the hair germ, at a site where transit amplifying cells are located [30], [31]. This restrictive *Lef1* expression is thought to allow proper compartmentalization [32]. In snakes, *Lef1* is mainly expressed in the highly proliferative successional lamina (this work, [17]). Interestingly, using DiI labelling we have shown that the successional lamina contributes cells to the next tooth generation, while a subset remains in the lamina. In summary, the dental epithelium appears to be organised into Sox2^+^ and *Lef1^+^*compartments. Further research is necessary to confirm the hypothesis that these regions contains stem and transit amplifying progenitor cells as observed in other regenerative organs.

### The Distal Tip of the Dental Lamina is *Lef1* Positive and Permits Polarized Elongation of the Dental Lamina

In snake tooth development the elongation of the dental lamina is essential to maintain dental tissue organization and polarity. We observed that *Lef1* expression is restricted at the free end of the dental lamina. It has been demonstrated that *Lef1* is expressed at the primordium tip of several organs that elongate. Expression of *Lef1* is localized at the leading edge of the zebrafish lateral line organ, which guides the migrating primordium allowing the deposition of organized and spaced neuromasts [33]. In addition, Wnt/β-catenin pathway activation is present in the distal tip of the epithelium of the extending lung primordium [34]. *Lef1* is also expressed at early stages of development in the presumptive epithelium that give rise to dental placodes [35] and controls the elongation of the dental lamina allowing the generation of inter-tooth spaces. Lef1 may be therefore part of a genetic module that was co-opted during animal evolution to elongate many different organs.

### Prolonged GSK3β Inhibition Increases the Number of Tooth Germs and Alters their Spacing, Directionality and Morpho-differentiation

In the snake, replacement teeth form from a permanent successional dental lamina. In the mouse, despite it being a monophyodont with no replacement teeth, the three molar tooth germs develop in succession in a comparable manner to the snake. However in the mouse, successional development stops after the formation of the third molars, while in the snake succession is continuous. Despite these differences, similarities can be made between the formation of replacement teeth in a polyphodont and the formation of successional teeth in a monophyodont, and it would be interesting to see whether similar signalling networks are involved in these two situations.

Here we examined the effect of continued activation of Wnt/β-catenin pathway in the dental organ of the snake using GSK3β inhibitors. We observed that LiCl and BIO increased the number of developing tooth germs and disrupted the normal polarity of tooth germ initiation. This effect was observed after GSK3β inhibition in both slice and block culture of the mandible. Our experiments were performed *ex vivo*. In the future it may be possible that new tools will allow us to confirm such phenotypes *in vivo*. A similar increase in the number of teeth was observed when Wnt/β-catenin was activated in the dental epithelium in the mouse [9], [10]. In animals that do not have multiple tooth replacement it is tempting to speculate that this could be controlled by changes in Wnt/β-catenin activation and localisation. To test this it would be interesting to locally increase Wnt/β-catenin signalling in the successional lamina in a monophyodont species to try and induce further generations. Such experiments are feasible and will allow a better understanding of how tooth number is controlled and can be manipulated.

We observed the extra tooth germs formed in the presence of GSK3β inhibitors were crowded, without directionality on succession and displayed defects in differentiation. Changes in *Lef1* expression appeared to underlie this phenotype, as Wnt/β-catenin pathway overactivation resulted in an expansion of the *Lef1* expression domain throughout the dental epithelium. The lack of a leading *Lef1* ^+^ edge was associated with an inhibition of the elongation of the dental lamina, with the formation of numerous closely associated epithelial projections. This resembled the phenotype of gain of function of Wnt/β-catenin signalling in APC zebrafish mutants where *Lef1* expansion produced close deposition of neuromasts along the lateral line [36]. Lack of a polarized expression of *Lef1* appeared to cause loss of directionality of the dental lamina, with new tooth germs budding from both the lingual and labial sides, rather than being restricted to the side of the successional lamina.

In addition to altering the expression domain of *Lef1*, inhibition of GSK3β led to the restriction of Sox2 to the oral dental epithelium. A similar phenotype, expansion of *Lef1* and restriction of Sox2 domains, was observed when Wnt/β-catenin pathway was activated in lung epithelium in the mouse [37].

We observed that the snake teeth from long-term cultures treated with GSK3β inhibitors failed to achieve their correct differentiation and shape. In the mouse, Wnt/β-catenin pathway activation also induced the formation of extra teeth with aberrant shape [9], [10], which have been likened to the formation of odontomas (odontogenic tumours). In our model we observed that prolonged activation of Wnt/β-catenin led to equivalent defects in epithelial invagination, with pathological changes in the epithelium similar to those observed in odontogenic tumours. In the BIO cultures hyperplasia and hypertrophy of the dental epithelium and tissue resembling ameloblastic fibroma was observed, characteristic of *Lef1* positive immature odontomas [38]. These experiments indicate that strict regulation of the Wnt/β-catenin pathway is necessary for tooth morphogenesis and to prevent the formation of odontogenic tumours.

In this work, the two different treatments to inhibit GSK3β, LiCl and BIO produce similar phenotypes, however BIO treated cultures had stronger effects than LiCl. This could be due to the fact that BIO is a more effective and specific inhibitor of GSK3β activity *in vivo*, with higher specific activity than LiCl [39]. The ability of GSK3β inhibitors to activate Axin2 and *Lef1* in our cultures supports overactivation of Wnt/β-catenin as a mechanism to increase the number of tooth germs. However, GSK3β is known to crosstalk with other signalling pathways [40], [41], and therefore the phenotype generated may not be exclusively due to activation of the Wnt/β-catenin pathway.

Taken together, prolonged GSK3β inhibition in snake tooth cultures impaired the successional pattern of tooth renewal as well as tooth morpho-differentiation, associated with changes in expression and to the morphology of the dental lamina.

### Prolonged GSK3β Inhibition Alters the Molecular and Proliferative Response of the Dental Epithelium and Disturbs the Organization of Tooth Germs

In our cultures, GSK3β inhibition was found to increase the proliferation of the entire dental organ. However, a higher increase in cell proliferation was observed in the oral dental epithelium, and aboral epithelium, compared to the enamel organ or successional lamina. Abundant mitosis within the oral dental lamina and the lack of elongation was associated with a shorter and wider lamina, and in some cases a double lamina. After GSK3β inhibition, changes in *Lef1* expression and proliferation were more prominent in the epithelium compared to the mesenchyme, suggesting that the epithelium directs the changes in tooth initiation. In the mouse, recombination experiments at early stages of tooth development have demonstrated that *Lef1* is required in the epithelium but not in the mesenchyme to induce tooth formation [35].

**Figure 6:**
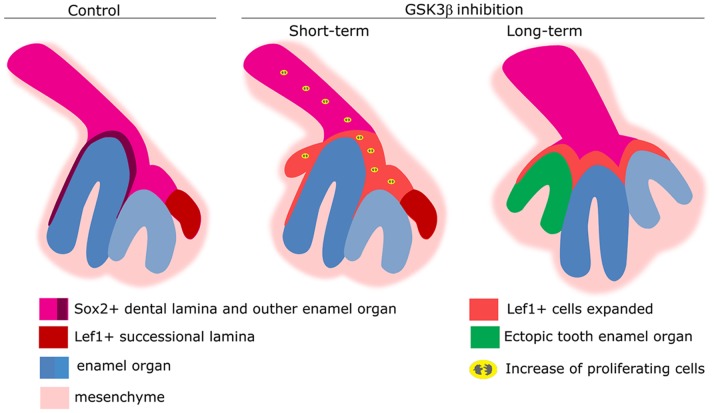
Working model for the emergence of tooth germs. The Wnt/β-catenin pathway controls the proliferative and molecular patterning of the snake dental lamina allowing the ordered emergence of tooth germs. In control cultures, Wnt/β-catenin signalling via *Lef1* is concentrated at the successional lamina (tip of the dental lamina). This region proliferates and elongates, regulating organized emergence of the tooth germs. In GSK3β inhibitor treatment *Lef1* expression is expanded and proliferation increases in the rest of the dental lamina, which becomes wider and shorter. The dental lamina becomes odontogenic and ectopic tooth germs and epithelial projections appear.

In our current working model, during normal development Wnt/β-catenin signalling is concentrated at the *Lef1* ^+^ successional lamina, with Sox2^+^ stem cells located slightly further back in the dental lamina. Active Wnt/β-catenin signalling leads to higher proliferation in the successional lamina, which elongates and produces the next generation of replacement teeth. When Wnt/β-catenin is activated throughout the dental primordium, *Lef1* expression expands and localised expression is lost. This results in an increase in proliferation in other parts of the dental epithelium, and the formation of ectopic tooth germs, as the dental epithelium acquires odontogenic potential. With the expansion of *Lef1,* the Sox2^+^ cells are shifted orally concentrating in the dental stalk, which expands as a consequence (Figure 6). Our results suggest that the compartmentalisation of cells at the tip of an elongating epithelium may be a conserved mechanism for the arranged emergence of sequential structures during development and regeneration.

## Supporting Information

Figure S1 **LiCl treatment increases the number of bud/tooth germs in snake mandible block cultures.** (A) Block cultured mandible. Tooth generations cannot be followed by this culture method but family tooth position can be identified as a circular area when viewed from the oral surface. Optical sections from (B) Control and (C) LiCl treated block cultures represented in A. Asterisks: dental lamina; white arrows: tooth germs; arrowhead: successional lamina. Addition tooth germs are observed on the labial side of the first generation tooth. (D) Graph showing the number of buds/tooth germs after 5 days in culture, paired between tooth families in the corresponding proximo-distal position. n = number of slices analyzed. Scale bars: (A) 500 µm, (B,C) 100 µm.
(TIF)
