# Supplementary figures and images for: Organized Emergence of Multiple-Generations of Teeth in Snakes Is Dysregulated by Activation of Wnt/Beta-Catenin Signalling
Source: PLoS One. 2013 Sep 3;8(9):e74484. doi: 10.1371/journal.pone.0074484 (PMC3760860; doi:10.1371/journal.pone.0074484)

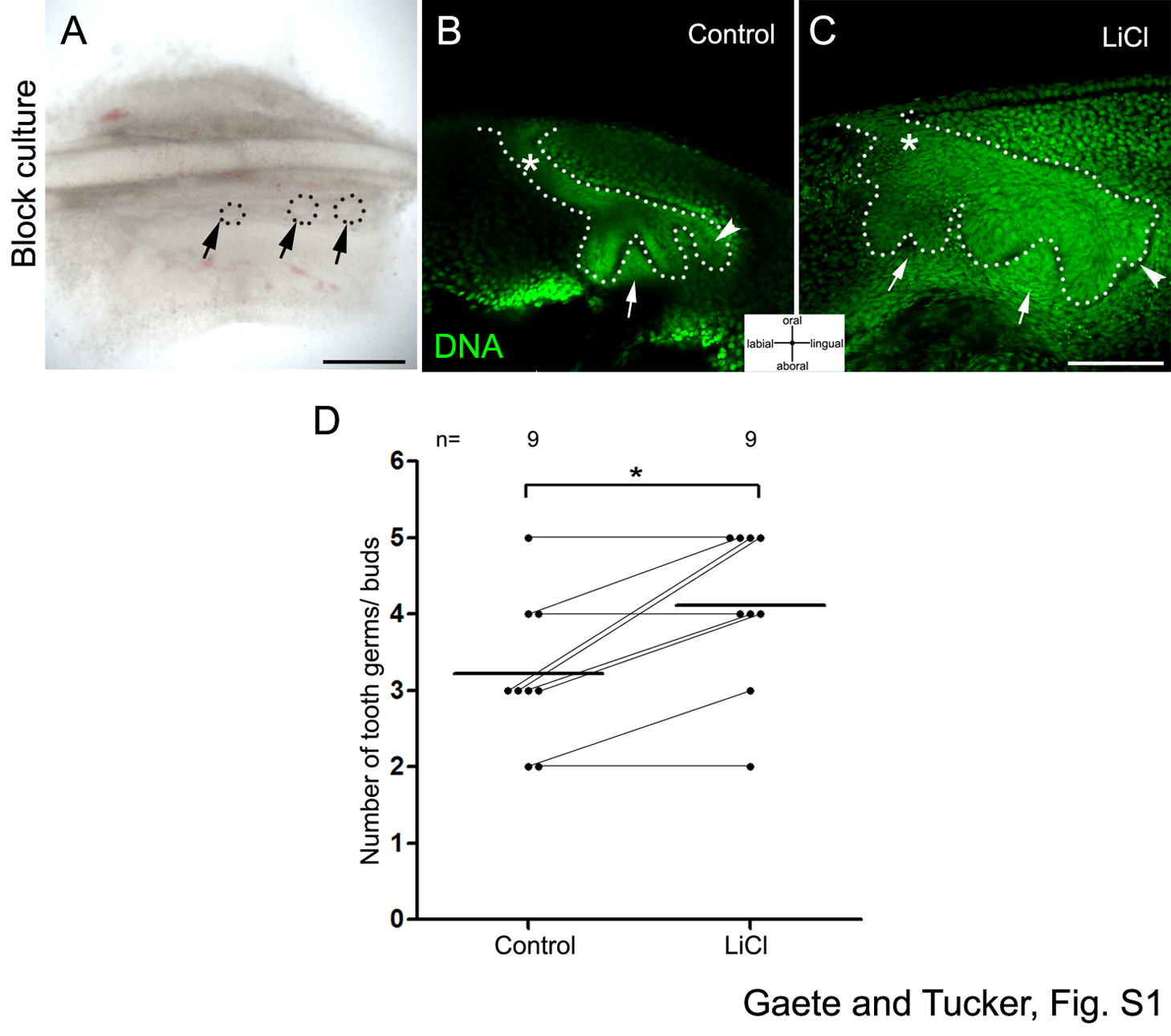

Supplement: Figure S1 — LiCl treatment increases the number of bud/tooth germs in snake mandible block cultures. (A) Block cultured mandible. Tooth generations cannot be followed by this culture method but family tooth position can be identified as a circular area when viewed from the oral surface. Optical sections from (B) Control and (C) LiCl treated block cultures represented in A. Asterisks: dental lamina; white arrows: tooth germs; arrowhead: successional lamina. Addition tooth germs are observed on the labial side of the first generation tooth. (D) Graph showing the number of buds/tooth germs after 5 days in culture, paired between tooth families in the corresponding proximo-distal position. n = number of slices analyzed. Scale bars: (A) 500 µm, (B,C) 100 µm. (TIF) [file pone.0074484.s001.tif]
